# Human behaviour and residual malaria transmission in Zanzibar: findings from in-depth interviews and direct observation of community events

**DOI:** 10.1186/s12936-019-2855-2

**Published:** 2019-07-01

**Authors:** April Monroe, Kimberly Mihayo, Fredros Okumu, Marceline Finda, Sarah Moore, Hannah Koenker, Matthew Lynch, Khamis Haji, Faiza Abbas, Abdullah Ali, George Greer, Steven Harvey

**Affiliations:** 1 PMI VectorWorks Project, Johns Hopkins Center for Communication Programs, Baltimore, MD USA; 2 University of Basel, Basel, Switzerland; 3 Swiss Tropical and Public Health Institute, Basel, Switzerland; 4 Environmental Health and Ecological Sciences Department, Ifakara Health Institute, Ifakara, Tanzania; 5 School of Public Health, Faculty of Health Sciences, University of the Witwatersrand, Parktown, Republic of South Africa; 6 Institute of Biodiversity, Animal Health and Comparative Medicine, University of Glasgow, Glasgow, UK; 7 Zanzibar Malaria Elimination Programme, Zanzibar, Tanzania; 8 U.S. President’s Malaria Initiative, U.S. Agency for International Development, Dar Es Salaam, Tanzania; 9 Department of International Health, Johns Hopkins Bloomberg School of Public Health, Baltimore, MD USA

**Keywords:** Malaria, Elimination, Residual transmission, Outdoor biting, Imported case, Migration, Travel, Human behavior, Qualitative research, Sub-Saharan Africa

## Abstract

**Background:**

Zanzibar has maintained malaria prevalence below 1% for the past decade, yet elimination remains elusive despite high coverage of core vector control interventions. As part of a study investigating the magnitude and drivers of residual transmission in Zanzibar, qualitative methods were utilized to better understand night time activities and sleeping patterns, individual and community-level risk perceptions, and malaria prevention practices.

**Methods:**

A total of 62 in-depth interviews were conducted with community members and local leaders across six sites on Unguja Island, Zanzibar. Twenty semi-structured community observations of night-time activities and special events were conducted to complement interview findings. Data were transcribed verbatim, coded, and analysed using a thematic approach.

**Results:**

Participants reported high levels of ITN use, but noted gaps in protection, particularly when outdoors or away from home. Routine household and community activities were common in evenings before bed and early mornings, while livelihood activities and special events lasted all or most of the night. Gender variation was reported, with men routinely spending more time away from home than women and children. Outdoor sleeping was reported during special events, such as weddings, funerals, and religious ceremonies. Participants described having difficulty preventing mosquito bites while outdoors, travelling, or away from home, and perceived higher risk of malaria infection during these times. Travel and migration emerged as a crucial issue and participants viewed seasonal workers coming from mainland Tanzania as more likely to have a malaria infection and less likely to be connected to prevention and treatment services in Zanzibar. Some community leaders reported taking the initiative to register seasonal workers coming into their community and linking them to testing and treatment services.

**Conclusions:**

Targeting malaria interventions effectively is critical and should be informed by a clear understanding of relevant human behaviour. These findings highlight malaria prevention gaps in Zanzibar, and the importance of identifying new approaches to complement current interventions and accelerate the final phases of malaria elimination. Development and deployment of complementary interventions should consider human behaviour, including gender norms, that can influence exposure to malaria vectors and prevention practices. Expansion of community-level programmes targeting travellers and seasonal workers should also be explored.

## Background

Wide-scale implementation of malaria interventions has led to significant reductions in malaria morbidity and mortality worldwide. The World Health Organization (WHO) estimates that between 2000 and 2015, the rate of new malaria cases declined by 37% globally, and malaria deaths fell by 60%, with 6.2 million lives saved [1]. An estimated three quarters of these gains can be attributed to core vector control interventions, specifically insecticide-treated nets (ITNs) and indoor residual spraying (IRS) [2]. However, progress is beginning to level off, with no significant changes in the number of malaria cases or deaths between 2015 and 2017 [3].

While achieving and sustaining high levels of coverage of core vector control interventions is essential, in many contexts, malaria can persist even once these targets have been achieved. A key challenge is the indoor-orientation of ITNs and IRS. Increases in outdoor vector feeding and resting in settings where people spend significant time outside at night may allow vectors to avoid interventions and consequently limit their effectiveness [4, 5]. An improved understanding of transmission that can persist in the context of high coverage of ITNs or IRS, referred to as residual malaria transmission, will be essential to elimination efforts [4–6].

In Zanzibar, the combination of effective vector control interventions with high quality diagnostics and case management led to a dramatic decline in malaria cases and deaths [7, 8]. ITNs were distributed across Zanzibar through universal coverage campaigns (UCC) in 2012 and 2016 with the goal of achieving one ITN for every two people. To sustain high coverage, ITNs have also been distributed continuously through health facility and community-based channels beginning in 2014 [9]. Demographic and Health Survey and Malaria Indicator Survey results show a high use to access ratio in Zanzibar, suggesting that people are largely using the ITNs they have [10]. In addition, focal spraying of IRS has been implemented based on village-level incidence data, with over 90% household coverage in spray sites each round [11].

Malaria parasite prevalence has been maintained below 1% for the past decade, and the islands can feasibly aim for elimination [12, 13]. In recent years, transmission, while low, has been geographically concentrated in locations with high vector abundance, and areas where residents and visitors frequently travel to malaria endemic regions outside of Zanzibar [14]. Results from entomological monitoring from 10 sentinel sites have shown a shift in the malaria vector population from *Anopheles gambiae* sensu stricto (s.s.) to *Anopheles arabiensis*, reflecting a shift toward outdoor biting patterns [15]. In some locations, *Anopheles funestus* and *Anopheles merus*, have also increased. Malaria vector density peaks during the long rains, occurring approximately from April through June [15].

Previous investigations of ongoing transmission have focused on monitoring the behaviours and infectiousness of malaria vectors and also on case detection and reporting. Despite growing evidence that malaria cases in Zanzibar are increasingly clustered in certain demographic groups or villages, less attention has been paid to night time activity patterns that may contribute to exposure. As part of a larger research study designed to better understand the magnitude and drivers of residual malaria transmission in Zanzibar, this study utilized qualitative research methods to explore night time activities and sleeping patterns, individual and community-level risk perceptions, and malaria prevention practices.

## Methods

### Study area

**Fig. 1:**
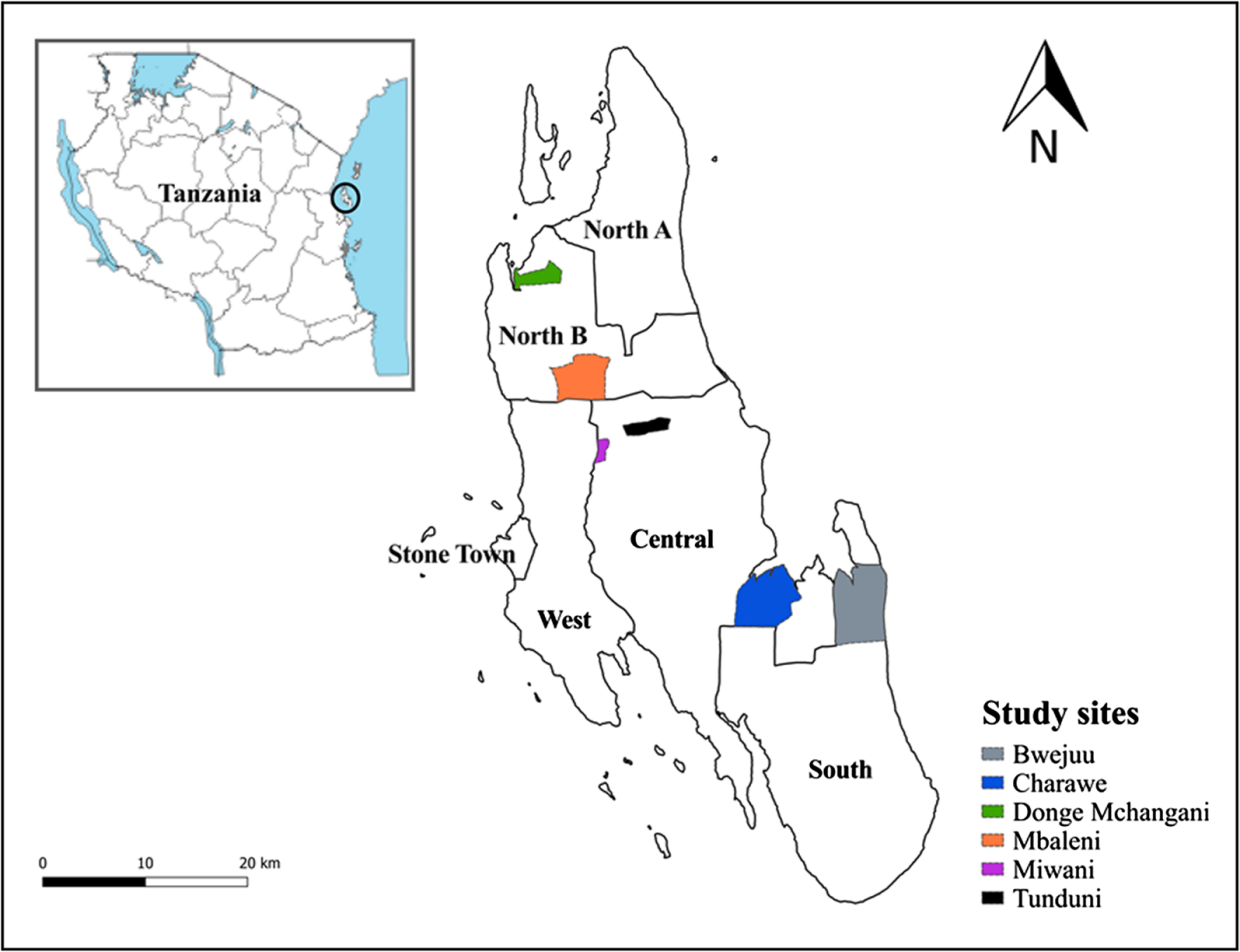
Map of study sites

This qualitative study was carried out in December 2016, during the dry season, and April–May 2017, during the long rains, across six *shehia* (wards) in Zanzibar. Sites were selected from across Zanzibar on the basis of high annual parasite incidence (API > 5/1000) and receipt of IRS in 2016. Selected sites were located on Unguja Island, the main island of Zanzibar, and included Bwejuu in the Southern District, Tunduni, Miwani, and Charawe in Central District, and Mbaleni and Donge Mchangani in North B District (Fig. 1). The study sites were all within 60 km from Stone Town, Zanzibar’s historical capital.

**Fig. 2:**
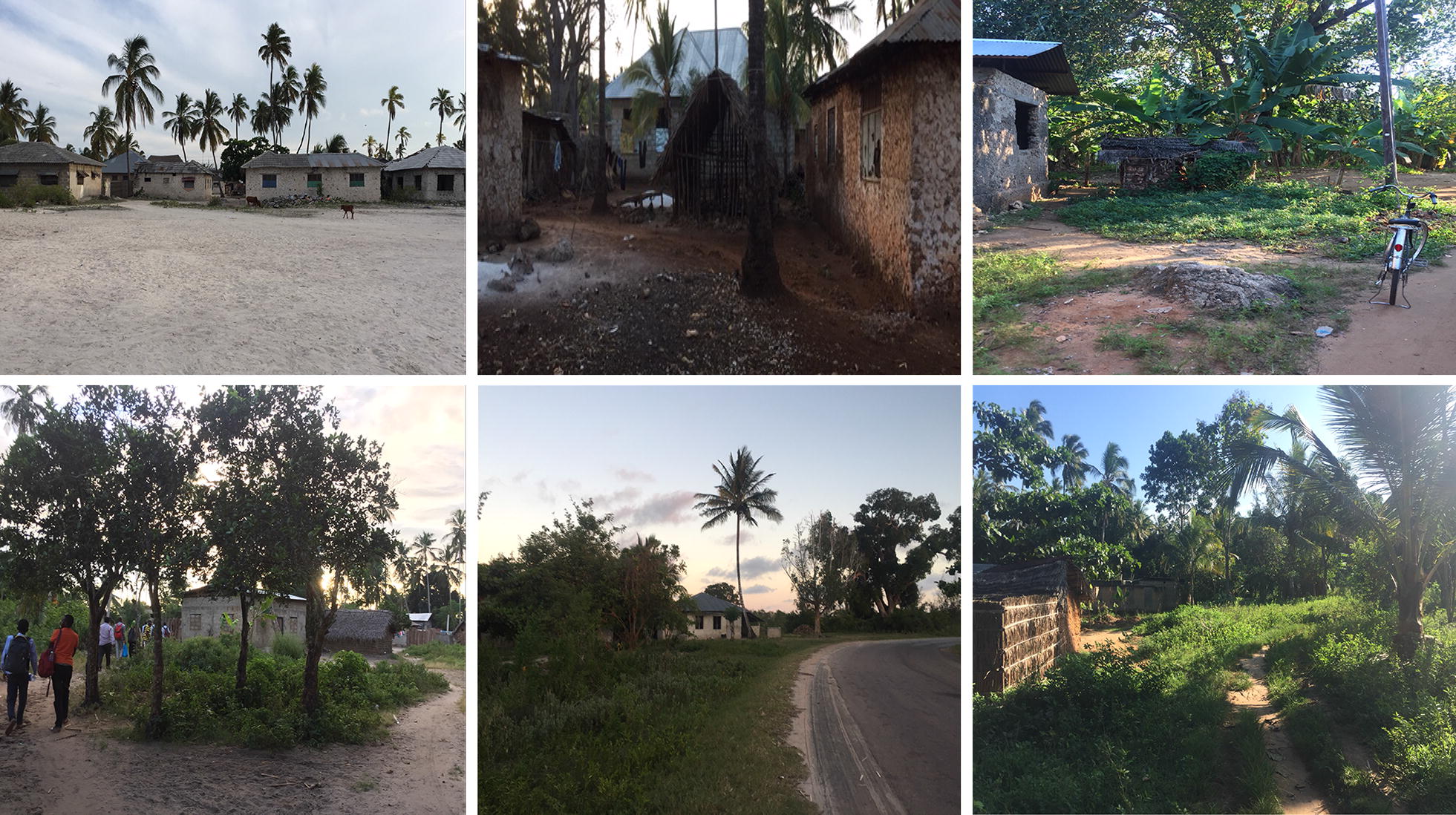
Photos of study sites. Clockwise starting from top left, the photos show Bwejuu, Charawe, Tunduni, Miwani, Mbaleni, and Donge Mchangani. Photos were taken during field work in December 2016 during the dry season

The selected *shehia* varied based on ecological features. Interior *shehia* such as Miwani and Tunduni, were characterized by lush vegetation, trees, and shrubs while Bwejuu and Charawe represented coastal communities (Fig. 2). Housing construction was both traditional and modern, and included materials such as brick and limestone, metal roofs, sticks and mud. Community members in the selected *shehia* participated in a variety of daily income-generating activities which ranged from fishing in the coastal areas, to farming, animal keeping, and small business activities. Community members were predominantly Muslim, however several communities, such as Miwani and Mbaleni, included a growing number of Christian residents from mainland Tanzania. Local leadership included the *Sheha* and *Assistant Sheha* who were elected to represent community members in each *shehia*.

### Study design

In-depth interviews (IDIs) were the primary method utilized, with supplemental night time community observations. Participants were eligible to participate if they were 18 years or older. For household IDIs, participants were drawn from among 135 randomly selected households included in the larger study. Households were selected using a random number generator from a list of households provided by the *Sheha* for each site. IDI participants were purposively selected from those households with the help of local leaders, to include representation from males and females of different ages from each site. IDIs were also carried out with community leaders, including the *Sheha* and his/her *Assistant Sheha* in each site, to better understand topics of interest at the community level and to identify night time activities and events occurring in outdoor public spaces.

Night time community observations were carried out to triangulate findings from in-depth interviews and to provide additional context for understanding community activities and events. Activities that took place away from home but within the study community, were identified with help from community leaders, who provided information on frequency, average attendance, and location.

### Data collection

#### Data collection team

The study employed data collectors from Zanzibar to ensure cultural understanding and sensitivity. Team members were selected on the basis of previous qualitative research experience, fluency in Swahili and English, and performance during training and pilot testing. The qualitative data collectors included three men and three women.

#### Community entry

Zanzibar Malaria Elimination Programme (ZAMEP) counterparts led a meeting to brief community and district-level leaders from the study sites, held at a central location, followed by visits to each study site. The community entry team worked with local leaders from each community to meet individually with heads of household for each of the selected households and explain the study objectives. At the beginning of the study, participants were briefed on the study objectives, and were notified by the *Sheha* or *Assistant Sheha* if they were selected to participate in an IDI.

#### In-depth interviews

IDIs were conducted using a semi-structured interview guide developed in English and translated into Swahili prior to data collection. Key topics of interest included night time activities and sleeping patterns, individual and community-level malaria risk perceptions, and malaria prevention practices. IDIs with community members were carried out in a quiet, private space, inside or close to the respondent’s home. IDIs with community leaders were carried out at the participant’s office or home. Interviews lasted 30–90 min. Data collectors audio-recorded all interviews and took field notes to capture detail on the setting and non-verbal cues. In-depth interviews were transcribed verbatim in Swahili by the data collection team, including non-verbal cues, then translated to English on an ongoing basis throughout data collection. Each data collector transcribed and translated his or her own interviews.

Team supervisors reviewed all Swahili transcripts and English translations and met with the qualitative data collection team on a weekly basis to review emergent themes, provide feedback on data quality, and discuss how to incorporate previous findings into subsequent interviews through an iterative process [16]. Follow-up interviews were carried out with a sub-set of community leaders to probe further on emergent themes of interest identified in the review process. Interviews were carried out until thematic saturation was achieved, defined as the point at which additional interviews no longer provided new information on the key topics of interest [17].

There were 62 in-depth interviews in total, evenly split across the six sites (Table 1). This included 44 household members, 13 community leaders, and follow-up interviews with five community leaders to explore emergent themes in greater detail.

**Table 1:** Interview participant demographics

	Number of interviews	Average age, in years
*Community members*	44	44.8 (range 24, 82)
Men	25	49.1 (range 30, 82)
Women	19	39.0 (range 24, 62)
*Community leaders*	13	51.9 (range 35, 71)
Men	10	54.3 (range 35, 71)
Women	3	43.7 (range 39, 48)
*Total respondents*	57	46.3 (range 24, 82)
Men	35	50.6 (range 30, 82)
Women	22	39.2 (range 24, 63)
*Follow-up interviews*	5	n/a
Men	4	n/a
Women	1	n/a
*Total interviews*	62	n/a
Men	39	n/a
Women	23	n/a

#### Night time community observations

Each night time community observation began with the *Sheha* or *Assistant Sheha* walking through the community with study team members to indicate the locations where routine community activities take place. The observers then walked to each location, ensuring at least one observation per location per hour. Data collectors carried out observations in pairs to ensure safety. They recorded the date, season, and location of each observation on a semi-structured form, then added a detailed description of the setting and activities every hour. Each hourly entry included a description of the activities ongoing at the location, approximate number people participating by age and gender, and use of malaria prevention measures, if any. The observation form also included space for the observer to document their own perceptions of what they saw in order to effectively capture both direct observation data and interpretations without mixing the two. For large-scale special events, the study team stayed at the location of the event from the time it began until the time it ended.

A total of 20 nights of community level observation were carried out, of which 17 were of routine activities, defined as activities that occur on a nightly basis. Routine observations were carried out across all sites at least once in both the dry and rainy seasons. Night time observations of routine community activities took place from approximately 6:00 p.m. until the final night time activity ended. The end time ranged from 11:05 p.m. to 3:45 a.m. for routine night time activities in the dry season, and 10:15 p.m. to 3:18 a.m. in the rainy season. Routine events observed included buying and selling at local shops, watching television in public spaces, and sitting at *Maskan*. *Maskan* originally referred to places where people of the same political party would go to meet, however the term is now used locally to describe any place where people, generally men, meet to socialize. Three special socio-cultural events were attended when the study team was notified by community leaders of an upcoming event. Large-scale special events were observed from the time they began until the time they ended, or until 7:00 a.m., whichever came first. The three large-scale events observed included a wedding ceremony, a religious ceremony referred to as *Dhikri*, which involved chanting throughout the night, and a rite of passage ceremony for adolescent girls.

Following each community observation, observers typed up field notes in English using a template mirroring the data collection form. All electronic files of field notes were collected and reviewed by team supervisors.

### Data analysis

Data was analysed through an iterative process, beginning during data collection through weekly reviews of data and emerging themes, as described above. Following data collection, members of the study team developed a preliminary codebook deductively based upon interview guides and research aims, and inductively through review of transcripts. The codebook included the code, a brief definition, an expanded definition, criteria for using the code, and examples of text from the transcripts to illustrate use of the code [18]. The study team tested the codebook on a random sub-sample of transcripts and met to discuss and review the coding exercise. The codebook was then finalized to reflect feedback from the discussion and to ensure a clear understanding of code definitions across all coders. All in-depth interview transcripts were then uploaded into ATLAS.ti 8, a qualitative data analysis software for ease of storage, indexing, and retrieval [19]. Members of the study team coded all transcripts in ATLAS.ti using the final codebook and used a thematic approach to data analysis [20].

### Ethical approval

This study received ethical approval from the Johns Hopkins Bloomberg School of Public Health (IRB# 7390), Ifakara Health Institute (IHI/IRB/No: 035–2016) and the Zanzibar Medical Research and Ethics Committee (Protocol #: ZAMREC/0005/OCT/016). All IDI participants provided written informed consent. The *Sheha* provided oral consent for community observations in public spaces. Heads of household provided written consent for photos. Results were presented back to ZAMEP and the Zanzibar Malaria Elimination Advisory Committee.

## Results

The results present information obtained through IDIs, with supplemental detail and context added from direct observation of community-level activities. Results are organized by theme and include night time activities and sleeping patterns, risk perceptions, malaria prevention practices, and travel and migration.

### Night time activities and sleeping patterns

**Fig. 3:**
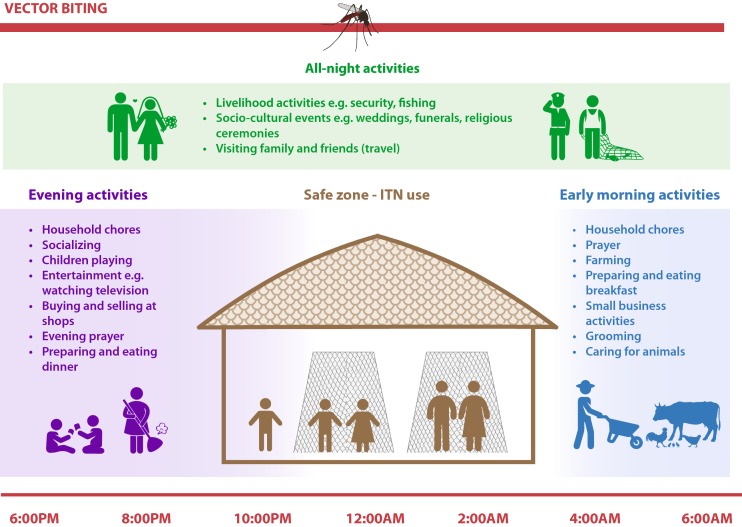
Illustration of common night time activities that occur during times when local malaria vectors are active. This includes routine activities in the evening and early morning as well as livelihood activities and special events that can last throughout the night

An illustration of night time activities occurring in the evening, early morning, and throughout the night is included in Fig. 3.

### Routine activities

In the peri-domestic space, in and near the home, many women reported doing household activities such as cooking, caring for children, and fetching water at wells in the early evening and at night. Participants also reported doing household chores in the early mornings; women prepared food and cleaned the house while men cared for animals.

**Fig. 4:**
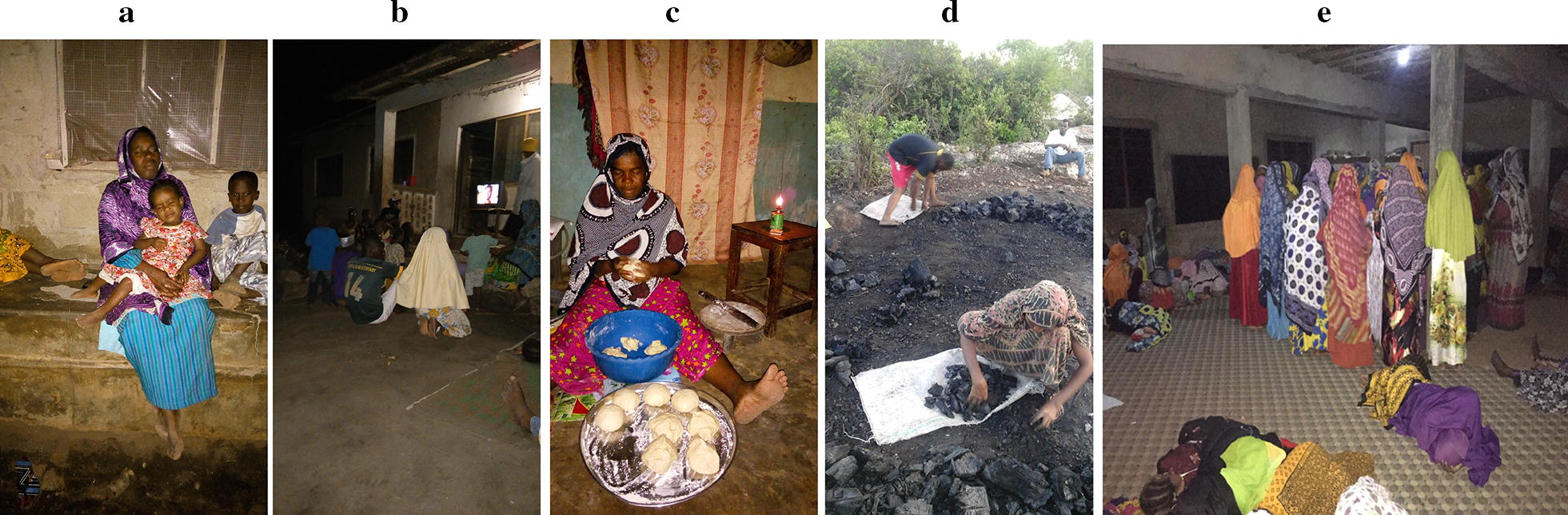
Photos of common night time activities. **a** A woman and her children sit outdoors on the verandah of their home; **b** community members gather outdoors to watch television; **c** a woman prepares *mandazi* (fried dough) indoors before bed to sell the next day; **d** a man and woman collect and package charcoal in the early evening; **e** women chant while others sleep on the floor during *Dhikri*, an all-night religious event

Away from home, small-scale social activities, ranging from two to twenty people, were widely reported and observed during the early evening and night time. Men gathered at *Maskan,* where they socialized and played cards or other games after finishing their day’s work. *Maskan* generally took place in the same locations each night, either outdoors or in semi-open spaces. Women and children were more likely to socialize outdoors nearby their houses with friends or neighbours (Fig. 4a).

Watching television or movies was another common social activity that occurred next to small shops or in other semi-covered areas such as market stalls or shacks. In some locations, only men watched television, while at other locations women and children were also observed watching, often in the early evening hours, from 7:00 to 9:00 p.m., compared to men who would frequently stay up to midnight. When football matches were being broadcast, young men stayed up to watch until the matches ended, sometimes until 2:00 a.m. or later (Fig. 4b). On weekends, youth were reported to go to *Viwanja* (entertainment sites, such as bars, that have drinking and dancing). These entertainment activities were most commonly reported in Miwani and Bwejuu and typically ended after midnight. Routine social activities were reported and observed to end earlier in the rainy season compared to the dry season. Football matches were an exception, as a female household member from Donge Mchangani explained, *“people who love football come regardless.”*

Buying and selling at local shops was commonly reported and observed. This included permanent shops selling packaged household goods, as well as small stalls selling *chipsi* (French fries), fish, bread, fruit and other foods. Both men and women engaged in small business activities. These shops generally opened in the early evening between 6:00 and 7:00 p.m. and closed by midnight (Fig. 4c). In the rainy season these activities were moved to semi-covered locations or closer to home, and shops generally closed earlier than in the dry season.

**Fig. 5:**
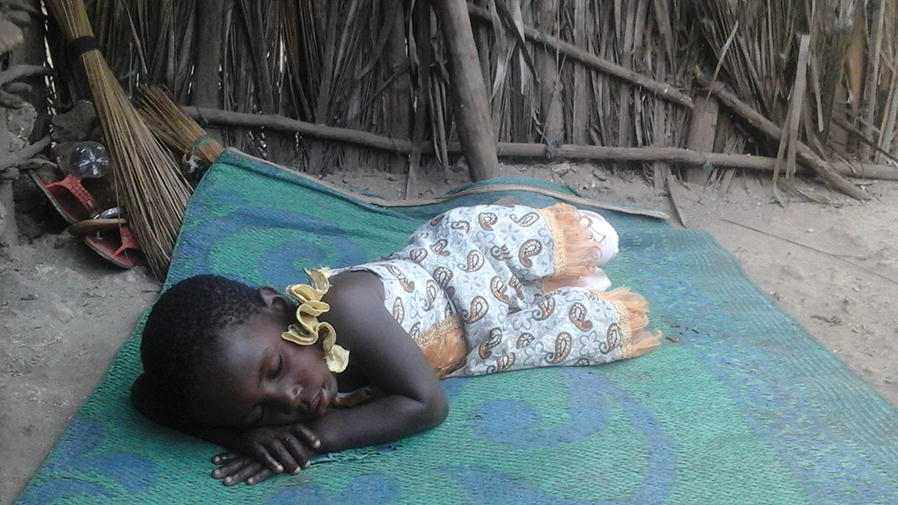
A child sleeps in an open-air space during early evening hours

During the dry season, some participants reported sleeping outdoors, largely because of the heat. Indeed, participants were observed dozing outdoors in the early night time at social gatherings, while watching television, or on verandahs and areas nearby house compounds (Fig. 5). Young men were observed sleeping outdoors more frequently during the evenings and night time, than women or children.

Across study areas, religious activities were routinely observed. Muslims prayed three times during the night; first, at sunset, then at approximately 8:00 p.m., and finally, at sunrise. Men generally prayed at the community mosque, while women were more likely to pray at home. Some older children, ages 10 to 14, attended *Madrasa* (Islamic religious school) during the evening hours, from 7:00 to 9:00 p.m. Religious activities continued throughout the year.

Livelihood activities, lasting all or part of the night, were commonly reported. In coastal communities, men made a living by fishing, while women and some children harvested seaweed. Along the coast, men often went fishing at night, sleeping in their boats at sea or in temporary shelters along the shore. Fishing activities took place year-round and the time of night depended on the tides and on the cycle of the moon. In some study areas, charcoal making was a common income-generating activity. It occurred away from the house, at designated locations within the community, and generally took several days to complete. This activity did not require someone to be outside continuously, but rather to intermittently check on the fires (Fig. 4d). Additional livelihood activities included security jobs, hunting, and working in hotels in coastal areas. In some locations, men reported staying outdoors to guard their crops from theft before harvest. A male household member from Mbaleni explained*, “They sleep at the area where they keep their stuff. For example, if you plant maize somewhere, you should stay [in] the same place to guard it so it can’t be stolen… and taken away by [thieves].”*

Many livelihood activities continued regardless of season. When asked about seasonal differences in livelihood activities, a male community member from Charawe explained, *“The work that people do, they do all the time. Everyone has to eat and make a living whether it’s the rainy or dry season.”* Farming activities intensified in the rainy season and some participants reported waking up earlier during this time in order to spend more of the morning hours farming.

### Special events

While routine activities, with the exception of livelihood activities, were reported and observed to generally finish by midnight, special events continued later, sometimes until dawn. Large-scale social events, including weddings, funerals, and religious ceremonies occurred year-round but were reported to occur more frequently in the dry season. These events were attended by all demographics, including men, women, and children. Events took place in large open outdoor spaces nearby or within household compounds. They brought together people throughout the community, and some attracted visitors from outside the ward.

At one wedding observed by study team members in the rainy season, a group of approximately 50 men and women danced in an open space, while a smaller group of approximately ten drank local brew nearby. The wedding began around 8:00 p.m. and lasted until 3:00 a.m. During heavy rains, events sometimes moved indoors. For instance, *Dhikri*, a religious event characterized by prayer and chanting, was scheduled to occur outdoors but was moved indoors because of rain (Fig. 4e). A few men remained outside cooking large pots of food under a small roof with no walls. The event lasted from approximately 10:00 p.m. until dawn and attracted men, women, and children from across multiple *shehia*.

Outdoor sleeping was also observed and reported during special events. Large numbers of people gathered and slept in or near the host’s home, such as on a verandah, or indoors in community structures such as a mosque with open doors and windows. Women and children were reported to sleep inside the host’s house while men were more likely to sleep outdoors.

While large-scale events typically lasted from one to several days, participants reported extended changes to sleeping and activity patterns throughout Ramadan, the Islamic holy month characterized by fasting from dawn until dusk. Participants reported staying up at night to pray, and spending some or all of the night sleeping outdoors.

#### Risk perceptions

When asked about the community’s most common health problems some participants mentioned malaria as a major concern, others reported it as minor, while others required prompting to mention it at all. Participants also described seasonal fevers but did not associate them with malaria. Participants frequently reported that malaria cases had largely decreased over the years. As a male key informant from Donge Mchangani explained, *“Malaria has decreased because in the past about 10 to 15 people would be diagnosed with malaria, but now only one to two people per month or sometimes no one, so malaria has decreased [to a large extent].”*

Men, women, and children were viewed as being at risk of malaria infection, but for different reasons. Men were perceived as being at risk due to their night time social and livelihood activities and travel, women were perceived as being at risk due to time spent outdoors doing household chores and socializing near the home, and children because of their inability to protect themselves. Amongst them, children were seen as being the most vulnerable and having the highest risk of severe malaria infection. A female household member from Charawe provided her view on what puts children at risk*, “They [children] stay and play near big puddles and bushes, and when you put them to bed under a net and leave, they can move to the sides of a bed and touch the net, so mosquitoes bite them.”*

When at home during the night time, study participants perceived that they were at higher risk of malaria infection outdoors than indoors. They described that it was easier to protect themselves from mosquito bites indoors because prevention methods such as ITNs, IRS, and aerosol insecticide sprays were available. However, a few participants noted that people could also be at risk indoors if they were not under ITNs.

A female household member from Donge Mchangani explained outdoor risk this way, *“when we watch TV (outside), we can spread a mat for children to sleep. Then you concentrate on the TV while the child is getting malaria through mosquito bites.”* In a similar way, participants perceived that they were at higher risk of infection when they were away from home (outside the *shehia*). This was largely attributed to lack of access to malaria prevention tools when visiting relatives, going to work, or attending special events away from home.

Alcohol consumption emerged as an important theme related to malaria exposure. It was discussed as a risk factor in three of the sites and was reported most frequently in Miwani. Study participants associated alcohol consumption with spending long periods of time outdoors during the night, and in some cases, falling asleep outside. Men were both observed and reported as more likely to drink. Drinking occurred both on a routine basis, in small outdoor gatherings and at bars, and at some large-scale social events, such as weddings.

Participants perceived a higher risk of biting and infection during the rainy season than the dry season. This was attributed to a greater density of mosquitoes during the rainy season caused by the presence of mosquito breeding sites from water collecting in puddles, ponds and debris (coconut shells, plastic, and garbage) when it rained. A smaller number of participants noted that while mosquito density was higher in the rainy season, risk may be heightened during the dry season due to high levels of outdoor sleeping and lower levels of ITN use.

#### Malaria prevention

Study participants viewed ITNs as the most important tool for protecting against mosquito bites and malaria infection. It was the primary prevention method participants reported using indoors and was perceived as offering protection both at the household and community level. Across study sites, participants reported high levels of ITN use, with some variation across seasons, sites, and demographic groups.

A female household member from Miwani noted lower levels of ITN use in her community due to lower mosquito density explaining, *“Many people protect themselves using mosquito nets, but in our village nowadays, there aren’t many mosquitoes here, so nets are just like decorations to us.*” Lower levels of ITN use were reported from participants in Miwani compared to other *shehia* suggesting differences in ITN use patterns across sites. Participants across *shehia* noted that men were less likely to use ITNs consistently, compared to women and children. One female household member from Donge Mchangani confided, *“It’s better to tell you the truth. Their father does not sleep under the net. I sleep under it with my child, but my husband does not, he says that with the net it is very hot… Some days he sleeps under the net but some he does not.”* Heat was a key barrier to ITN use in the dry season and was reported across participants and sites. As one male household member from Donge Mchangani described, *“During the hot season everything is hot, even the net itself, if you are covered with a net you’ll be on fire.”*

Alternative prevention methods that were widely available included aerosol insecticide sprays and insecticidal coils. Participants reported purchasing these at local shops for use at home, with sprays largely used indoors and coils used primarily outdoors or in semi-open spaces.

Many participants noted the difficulty of malaria prevention outdoors. A female household member from Bwejuu described the challenge this way, *“When you are outside you really can’t wear the bed nets, can you?”* While one community leader reported seeing an example of outdoor ITN use, in general, outdoor prevention methods were either less known or not available. For instance, there was strong interest in topical repellents, especially for use when people were away from home working, visiting neighbours, or socializing. However, participants reported that topical repellents were not widely available in the community and were only sold at shops in town.

Some study participants reported that cost was a key barrier in accessing repellents, insecticidal sprays, and other alternative malaria prevention methods. Unlike ITNs, which were distributed freely, other methods were only available for purchase at local shops. In some study areas participants mentioned using traditional repellents, made from local plants, that were less expensive. These included tablets that produced mosquito repelling incense (similar to insecticidal coils), and lotions that were applied when outdoors.

Heads of households were primarily responsible for making decisions on purchasing or using malaria prevention methods in the household. Parents reported making decisions in the best interest of their children and were responsible for providing additional or alternative prevention methods if current methods were insufficient, for example, replacing ITNs when they were torn/worn out and buying insecticidal spray to prevent indoor biting.

Housing quality was seen as a key determinant of malaria transmission. Open eaves, unfinished houses, and lack of window screening were viewed as contributing to increased malaria risk. Cost was frequently listed as the key barrier to housing improvements. However, in spite of challenges in housing quality, window screening was commonly reported and observed in the study sites. Window screening served a dual purpose of malaria prevention and providing extra privacy and security. A male household member from Donge Mchangani highlighted the security value, saying*, “If you haven’t covered your windows there are thieves, [someone] can put their hand inside if something is nearby and steal it, but screening provides that protection, when you cover (the window) with wire, it is not easy for things to be stolen.”*

Education was viewed as a critical component of malaria prevention. Participants described that education on malaria prevention was given at the health facility, through community meetings, and before ITN distribution and IRS campaigns. Community leaders also played an important role in educating community members. Participants reported being satisfied with the level of education provided, but noted that frequent and consistent education, particularly for non-users of ITNs, is crucial.

#### Migration and travel

Migration and short-term travel were viewed by participants as a critical challenge to malaria elimination in Zanzibar. Participants reported travelling to mainland Tanzania to work, visit family, and for special events such as weddings and funerals. In some study sites, there had also been an influx of migrants and visitors coming to work from across Zanzibar, mainland Tanzania, and other parts of East Africa. Seasonal workers were reported to come for farming, to operate small businesses, and work in hotels in coastal areas. Some came seasonally, for a few months, while others stayed for longer periods, even settling permanently.

Increased presence of migrants in study areas was described as being responsible for imported cases as well as subsequent local infections. A male household member from Bwejuu explained*, “There’s an influx of people from outside coming into this town…There’s almost as many people from outside here as there are locals, and that makes it worrisome.”* Additionally, visitors were perceived as less likely to be linked to community channels for malaria treatment and prevention such as the health facility, community health committees, or community leaders. Seasonal workers and other visitors were likewise viewed as less likely to have access to or use ITNs that are available to other members of the community, making them more vulnerable to infections. Both household members and key informants noted the importance of providing ITNs to visitors. A male household member from Donge Mchangani explained, *“We interact with our friends from Tanzania mainland and they come more and more every day and they need nets.”*

Community leaders in three study sites reported examples of how these challenges were already being addressed, or how they planned to address them in the immediate future, through initiatives spearheaded at the *shehia* level. In these sites, visitors will be required to report to the *Assistant Sheha* and *Sheha* when they arrive and get tested for malaria as a prerequisite for living and working in the *shehia*. Key informants in one site reported that this initiative has already been implemented and has led to identification and treatment of positive malaria cases.

## Discussion

Targeting malaria prevention interventions requires an understanding of when and where people are being exposed to malaria vectors. These results provide a richer understanding of human behavioural factors that can impact exposure risk and inform malaria elimination efforts in Zanzibar.

Zanzibar has one of the most intensive and successful programmes for malaria control and elimination in sub-Saharan Africa, including wide-scale implementation of effective vector control tools, case management, and social and behaviour change communication. While the islands have experienced significant reductions in both vector density and malaria infection, current vector control interventions, namely ITNs and IRS, may not be sufficient for eliminating local transmission [21].

This study revealed that in Zanzibar, as in other settings in sub-Saharan Africa, a range of routine activities occur during times when malaria vectors are active [22–25]. A 2010 study by Dunn et al. described malaria risk behaviours in rural Tanzania and established that routine social, household and livelihood activities around dawn and dusk including socializing outdoors, fetching water, fishing, and farming could increase risk of exposure to mosquito bites and infection [24].

Large-scale socio-cultural events and livelihood activities were identified that lasted throughout the night, disrupting usual sleeping patterns and making ITN use during any part of the night a challenge. This finding is consistent with a number of recent studies highlighting the importance of ‘special’ socio-cultural events in malaria-related risk [22–24, 26]. Studies in Ghana and Uganda identified social barriers to ITN use away from home such being perceived as proud or even disrespectful for using ITNs during important social events. They also identified logistical constraints, including not having a place to hang an ITN at social events, and resource limitations, such as not having extra ITNs with which to travel [22, 23].

To date, there are few tools designed to protect people outdoors that have sufficient evidence to support a WHO recommendation. Tools that are in the development pipeline should address existing gaps in protection and be appropriate to the context in which they will be used. The results suggest discrete times of the year and setting profiles that can help to inform development and deployment of supplemental interventions. This includes inside the home, directly outside of the home, fixed locations within the community where people routinely gather, large-scale events, livelihood activities, and travel.

Understanding how gender norms impact risk of exposure to malaria will also be essential for targeting vector control tools and social and behaviour change interventions [24, 27]. In Zanzibar, women were more likely to serve as primary caregivers, and perform daily tasks that were oriented around the household, including childrearing, household chores, and operating small businesses close to home such as selling food and charcoal making. Women with children also stayed closer to the house during the night time, often socializing with neighbours and friends near their home.

Conversely, men’s routine activities had a higher level of physical mobility, keeping them outdoors and away from their home for longer periods during the night time. Across seasons, men stayed outdoors later and went to sleep later after spending the night watching television, chatting, and socializing at *Maskan*. In some sites, men also reported drinking alcohol, which was perceived to increase risk behaviour, such as staying outdoors late into the night. Men were more likely to engage in night time livelihood activities, including security jobs, protecting crops, hunting, and fishing which keep them outdoors throughout the night. These findings are consistent with a trend seen in many countries moving toward elimination where adult males represent a rising proportion of malaria cases [28]. This trend has been largely influenced by migration and outdoor livelihood activities that increase exposure to malaria vectors, including fishing, agriculture, military, mining, and forest work [29].

Travel and migration emerged as a central consideration for malaria elimination in Zanzibar among study participants. This finding is consistent with a recent review of travel history among malaria cases in Zanzibar which showed more than half of cases had travelled outside of Zanzibar in the previous month [15]. In this study, many residents perceived that travellers, returning residents, and seasonal workers from Mainland Tanzania are responsible for new malaria cases, representing a key threat to malaria elimination in Zanzibar. Even when residents perceived overall malaria transmission rates to be low, they believed that there was a risk of resurgence of malaria due to travel and migration. These results are consistent with the findings of a 2012 study in Zanzibar in which participants expressed a fear that imported cases from the mainland could put local communities at risk [30]. Research across a number of settings in sub-Saharan Africa have documented an association between travel from higher to lower transmission areas and malaria risk [31–33], and available evidence suggests that targeting imported cases is pivotal to malaria elimination efforts [34].

Livelihood activities and occupations such as tourism, farming, and fishing were seen as key drivers of short-term migration. For instance, farmers from mainland Tanzania moved into some study sites to farm and cultivate land often arriving during planting season and staying through harvest season when they were paid for their work. Participants also perceived that seasonal workers had lower access to prevention methods that were available to residents such as ITNs distributed through community channels. Seasonal workers and migrants were seen as less likely to seek health care resources, and more likely to engage in night time occupations and activities that put them at higher risk of infection.

Participants in this study noted that community level malaria prevention programmes often missed or were not accessible to seasonal workers and migrants. These included ITN distribution programmes and community-level education on malaria prevention. In some study sites, seasonal workers and visitors were required by the *Sheha* to register and get tested as a requirement for staying in the community. These community-level initiatives have the potential to ensure prompt testing and treatment for groups at higher risk of malaria infection. Complementary approaches for elimination should therefore include efforts to protect the migrant workforce, travellers, and guests from infections and ensure improved access to prompt diagnosis and treatment for these groups. ZAMEP could consider formalizing and expanding community-level programmes to other sites with high malaria incidence and high levels of seasonal workers and travellers, while maintaining leadership at the community-level. Additional interventions tailored to these groups could also be explored such as community health programmes encouraging residents to travel with ITNs, testing and treatment for seasonal workers, and providing a basic package of interventions for high-risk travellers. This approach is consistent with approaches being used in malaria elimination contexts in the Greater Mekong Sub-Region, where the current WHO malaria elimination strategy emphasizes the importance of increasing access to diagnostic and treatment services and prevention measures for mobile and migrant populations [35].

## Limitations

Qualitative research has inherently different objectives and methods compared to quantitative research [36], and it is crucial to interpret all study results in the context of the strengths and limitations of the methods used. The sample size in this study was designed to achieve theoretical saturation and not wide-scale generalizability. Further, self-reported information can be subject to bias, including social desirability bias in which participants provide responses they believe will be socially acceptable rather than responses that reflect their true experiences or perspectives [37]. To reduce the potential for social desirability bias, the study team conducted all interviews in private settings and reminded participants that there was no right or wrong answer to questions. Direct observation creates the potential for reactivity, a phenomenon in which participants alter their normal behaviour in response to the presence of an observer [38]. A few cases of reactivity, such as participants talking to or engaging with observers, were carefully recorded, and generally appeared to have a minimal impact on study results. Notwithstanding the limitations, these findings provide deeper insight into participants’ perspectives and experiences that would not be possible through more structured methods. Further, the combination of in-depth interviews and direct observation allowed for triangulation of information across methods.

## Conclusions

In Zanzibar, and other pre-elimination settings, targeting interventions effectively is critical, and should be informed by a clear understanding of relevant human behaviour. Gaps in malaria prevention identified in this study included routine household and community activities occurring during non-sleeping hours as well as livelihood activities and large-scale social events that often last throughout the night. There is an opportunity to explore the use of supplemental vector control tools and accompanying social and behaviour change interventions to help address these gaps. Migration and travel of malaria infected individuals from higher transmission settings into Zanzibar must also be addressed to reach elimination. Existing community structures provide potential mechanisms for addressing gaps in protection, as well as targeting higher risk groups, such as travellers and seasonal workers. Building on these existing systems to target interventions should be explored to limit both local and imported malaria cases.

## Data Availability

The datasets used and/or analysed during the current study are available from the corresponding author on reasonable request.

## Abbreviations

API: annual parasite incidence
IDI: in-depth interview
IRS: indoor residual spraying
ITN: insecticide treated net
WHO: World Health Organization
ZAMEP: Zanzibar Malaria Elimination Programme
